# Malaria Prevention with IPTp during Pregnancy Reduces Neonatal Mortality

**DOI:** 10.1371/journal.pone.0009438

**Published:** 2010-02-26

**Authors:** Clara Menéndez, Azucena Bardají, Betuel Sigauque, Sergi Sanz, John J. Aponte, Samuel Mabunda, Pedro L. Alonso

**Affiliations:** 1 Barcelona Centre for International Health Research (CRESIB), Hospital Clinic, Institut d'Investigacions Biomedicas August Pi i Sunyer (IDIBAPS), Universitat de Barcelona, Barcelona, Spain; 2 The Manhiça Health Research Centre (CISM), Manhiça, Mozambique; 3 National Directorate of Health, and National Institute of Health, Ministry of Health, Maputo, Mozambique; 4 National Malaria Control Program, Ministry of Health, Maputo, Mozambique; Mahidol University, Thailand

## Abstract

**Background:**

In the global context of a reduction of under-five mortality, neonatal mortality is an increasingly relevant component of this mortality. Malaria in pregnancy may affect neonatal survival, though no strong evidence exists to support this association.

**Methods:**

In the context of a randomised, placebo-controlled trial of intermittent preventive treatment (IPTp) with sulphadoxine-pyrimethamine (SP) in 1030 Mozambican pregnant women, 997 newborns were followed up until 12 months of age. There were 500 live borns to women who received placebo and 497 to those who received SP.

**Findings:**

There were 58 infant deaths; 60.4% occurred in children born to women who received placebo and 39.6% to women who received IPTp (p = 0.136). There were 25 neonatal deaths; 72% occurred in the placebo group and 28% in the IPTp group (p = 0.041). Of the 20 deaths that occurred in the first week of life, 75% were babies born to women in the placebo group and 25% to those in the IPTp group (p = 0.039). IPTp reduced neonatal mortality by 61.3% (95% CI 7.4%, 83.8%); p = 0.024].

**Conclusions:**

Malaria prevention with SP in pregnancy can reduce neonatal mortality. Mechanisms associated with increased malaria infection at the end of pregnancy may explain the excess mortality in the malaria less protected group. Alternatively, SP may have reduced the risk of neonatal infections. These findings are of relevance to promote the implementation of IPTp with SP, and provide insights into the understanding of the pathophysiological mechanisms through which maternal malaria affects fetal and neonatal health.

**Trial Registration:**

ClinicalTrials.gov NCT00209781

## Introduction

Every year over four million babies die in the first four weeks of life, most of these deaths, - three million - occur in the first week of life. Of this mortality, 99% take place in developing countries, where the risk of death in the neonatal period is six times greater than in developed countries. [1] [2] [3] [1], [2] [3] With more than 40 neonatal deaths per 1000 live births, the risk of dying during the neonatal period is highest in sub-Saharan Africa. [1], [2] Lowering child mortality increasingly depends on tackling neonatal mortality. Reducing neonatal deaths requires knowing its causes. Globally, it is estimated that prematurity, infections and asphyxia are the main direct causes of neonatal death. [3] However, in developing countries where the majority of these deaths occur, establishing the causes of neonatal mortality has an inherent uncertainty due to the limited quality and quantity of the sources of information. Moreover, few studies, either descriptive or on neonatal interventions focus on the deaths occurring in low income countries, and thus, many neonatal deaths still occur without an obvious cause. [3]

There are little doubts that public health interventions such as immunization, improved nutrition, water and sanitation have contributed to child survival^1^. However, maternal and neonatal survival requires additional and specific interventions and approaches. These should be based on an improved knowledge of their causes and determinants. [4] Malaria infection during pregnancy has been mentioned as one of the contributors to neonatal mortality, mostly through low birth weight (LBW) and by causing maternal anemia. [5] Although this extrapolation is probably correct, a direct association between maternal malaria and neonatal mortality has not yet been confirmed. This knowledge would help to improve the understanding of the physiopathological mechanisms of the effect of maternal malaria on the newborn and infant, as well as to promote the implementation of preventive interventions.

For malaria stable transmission areas in Africa, where approximately 25 million pregnancies are exposed every year to the infection, WHO recommends preventive strategies during pregnancy. [6] These measures are based on the administration of intermittent preventive treatment (IPTp) and the use of insecticide treated nets (ITNs). [6] IPTp consists on the administration of at least two doses of sulphadoxine-pyrimethamine (SP), regardless of the presence of parasites. The currently recommended regimen for IPTp is at least 2 treatment courses of SP given from the 2^nd^ trimester onwards at least one month apart.

The uptake of these tools varies between countries but it is still far from covering the majority of the pregnant women at risk of malaria in Africa. [7], [8], [9] To date, there is no confirmatory evidence from the published malaria prevention trials in pregnancy of a significant effect of the interventions on infant survival. [10], [11], [12], [13], [14] The lack of conclusive information has probably affected resource prioritisation for malaria control in pregnancy in many African countries.

We have recently reported the results on maternal and birth outcomes of a randomised, double blind, placebo-controlled trial of IPTp in Mozambican pregnant women (trial registration number: NCT.00209781). In this trial, it was found that IPTp with SP was associated with a moderate but significant reduction in the incidence of clinical malaria during pregnancy, and with a statistically significant reduction in the prevalence of parasitaemia at delivery and at 8 weeks postpartum, as well as in fetal anemia in cord blood. On the other hand, there were no significant differences in the prevalence of low birth weight, prematurity nor maternal anemia at delivery between the two groups. [15] The study also assessed the impact of the intervention on infant's survival. Here we present the effect of malaria prevention with IPTp on survival during the first year of life.

## Methods

### Ethics Statement

The study protocol was approved by the National Mozambican Ethics Review Committee, and the Hospital Clinic of Barcelona Ethics Review Committee. The protocol for this trial and supporting CONSORT checklist are available as supporting information; see Checklist S1 and Protocol S1.

### Study Area

The study was carried out at the Centro de Investigação em Saúde da Manhiça (CISM) in Manhiça District, southern Mozambique. A demographic surveillance system (DSS) covering around 80 000 inhabitants is carried out by the CISM and constitutes the main study area. The demographic and malaria epidemiological characteristics of the area have been described in detail elsewhere [16]. Malaria transmission of moderate intensity is perennial with some seasonality. More than 95% of the malaria infections are due to *P. falciparum*. [17] The estimated entomological inoculation rate for 2002 was 38 infective bites per person per year. [18] Data on the efficacy of SP in children in this area showed a therapeutic efficacy rate of 83%, with an *in vivo* parasitological sensitivity of 78.6% at day 14. [19] Adjacent to the CISM is the Manhiça District Hospital (MDH), a 110 bed health facility. More than 80% of the deliveries in the area are institutional. At the time of the study, the prevalence of HIV infection at the antenatal clinic was 23.6%. [15] Infant (IMR) and neonatal (NMR) mortality rates for 2005–2006 in the area were 75 and 26 per 1000 live births respectively (Nhacolo A., personal communication). During the study, malaria control in pregnancy relied exclusively upon case management. Since October 2005, the current Mozambican policy to control malaria in pregnant women is also based on ITNs and IPTp administration.

### Study Design and Follow Up

From August 2003 to April 2005, 1030 pregnant women of all gravidities were enrolled at the MDH into a double blind, randomised, placebo-controlled trial of two-dose IPTp with SP (IPTp-SP). Women were randomised to receive three tablets of SP (1500 mg sulphadoxine/75 mg pyrimetamine) or placebo. At recruitment all women received a LLITN. The primary objective of the study was to estimate the additive protective effect of IPTp-SP to that of ITNs on LBW prevalence. At the time of delivery, the baby's birth weight was measured on a digital scale and gestational age was assessed by the Dubowitz's method. [20] Maternal peripheral and cord blood were collected and a placental biopsy was taken. As an exploratory objective was the assessment of the impact of the intervention in the mother on the health of the child during the first year of life.

Maternal, birth outcomes and clinical information up to eight weeks post delivery have been already reported. [15] After the code was broken in 2006 two months after the last delivery, the trial was formally considered unblinded. However, all study personnel involved in the collection of the trial's outcomes was not aware of the participant's study group.

We present here a full analysis of the effect of the intervention on infant's survival up to the last child born to the study women reached one year of age. Infants were followed up through regular home visits to assess resident status until the child was one year of age.

A discrepancy in the number of neonatal deaths with those already reported [15] was observed when doing the final analysis once the study followed up was completed. A correction to the number of deaths has been submitted to the original journal (22^nd^ January 2010).

### Statistical Methods and Definitions

Neonatal mortality was defined as the death of a live born baby within the first 28 complete days after birth. Early neonatal mortality was defined as the death of a baby during the first seven days of life, while late neonatal mortality referred to deaths occurring after the 7^th^ day but before 28 complete days of life. [21] Infant mortality was defined as deaths occurring during the first 12 months of life. Malaria infection in the placental tissue was classified as past (presence of malaria pigment only), active (presence of parasites with or without malaria pigment), and not infected (neither parasites nor malaria pigment). [22]

Data were analysed by intention-to-treat (ITT) analysis, whereby infants born to all randomised women were included regardless of whether or not the mother had received the intervention during pregnancy and the number of doses taken. Only live born babies (single and multiple deliveries) were included in this analysis.

Incidences were calculated using time at risk from date of birth until date at one year of age, death or withdrawal. Incidences were expressed as episodes per PYAR where PYAR is Person Years at Risk. The protective efficacy (PE) of SP was estimated from the hazard ratio (HR) as PE = 100(1-HR)%. Association between risk factors and death was evaluated using Cox Regression Models adjusted by the intervention group. Variables for the multivariate analysis were selected using the backward -stepwise elimination procedure with a p-value less than 0.05. Differences in proportions were estimated with the Fisher's exact test. Continuous values were evaluated with the non-parametric Wilcoxon test. Missing values were categorized as unknown and their proportion was similar in the two intervention groups. Data analysis was performed using Stata 10 (Stata Corporation, College Station, TX, USA).

## Results

Demographic status at one year of age was documented for 997 of the 1004 live born babies (99%) to the 1030 enrolled pregnant women (500 were born to women who were in the placebo group and 497 to women in the IPTp-SP group) (Figure S1). The number of multiple deliveries was similar between the two groups (p = 0.2).

There were a total of 58 infant deaths. Of them, 35 (60.4%) (IMR 70 per 1000 live births) were babies born to women who had received placebo during pregnancy and 23 (39.6%) (IMR 46.3 per 1000 live births) were born to women who had received IPTp-SP (p = 0.136) (Table 1).

**Table 1:** Neonatal and infant mortality by intervention group.

		Intervention groups						
		Placebo+ITNs		SP+ITNs		Total		p
		n = 500		n = 497		n = 997		
**Neonatal mortality (%)** (≤28 days of life)	**Alive**	482	(96%)	490	(99%)	972	(97%)	0.041
	**Deaths**	18	(4%)	7	(1%)	25	(3%)	
**Early neonatal mortality (%)** (≤7 days of life)	**Alive**	485	(97%)	492	(99%)	977	(98%)	0.039
	**Deaths**	15	(3%)	5	(1%)	20	(2%)	
**Infant mortality (%)** (first 12 months of life)	**Alive**	465	(93%)	474	(95%)	939	(94%)	0.136
	**Deaths**	35	(7%)	23	(5%)	58	(6%)	

SP = sulphadoxine-pyrimethamine. ITN = insecticide-treated nets.

There were a total of 25 neonatal deaths. Of them 18 (72%) (NMR 36 per 1000 live births) babies were born to women in the placebo group and 7 (28%) (NMR 14 per 1000 live births) were born to women who had received IPTp-SP (p = 0.041) (Table 1).

Of the 25 babies who died within the 28 days after birth, 20 (80%) died within the first week of life (early neonatal mortality). Of these 20 deaths, 15 (75%) were among babies whose mother received placebo during pregnancy and 5 (25%) among babies whose mothers received IPTp-SP (p = 0.039) (Table 1). Among the children who died in the first week of life, mean age at death in the placebo group was 1.4 days (SD 2.1) compared to 2.8 days in the SP group (SD 3.0) (p = 0.4).

Only nine of the 25 newborns who died in the neonatal period died at hospital, and the cause of death could be ascertained. In all, except one, an infectious disease was the most likely cause of death. Of these, six were children born to women in the placebo group and two were to those in the IPTp-SP group.

IPTp-SP was associated with a statistically significant reduction in neonatal mortality [PE 61.3% (95% CI 7.4%, 83.8%); p = 0.024]. The effect of IPTp-SP on neonatal mortality did not vary by parity [PE 61.9% (95% CI 8.8%, 84.1%); p = 0.022] or HIV infection [PE 62.7% (95% CI 10.5%, 84.4%); p = 0.020]. The intervention had a non-statistically significant protective efficacy in reducing infant mortality by 35.2% [(95% CI −9.6%, 61.7%); p = 0.102] (Table 2).

**Table 2:** Protective efficacy of IPTp with SP on neonatal and infant mortality by intervention group.

	Intervention group							
	Placebo+ITNs			SP+ITNs			Protective Efficacy	
	Events	PYAR	Incidence	Events	PYAR	Incidence	% (95% CI)	p
**Neonatal mortality** (≤28 days of life)	18	37.3	0.48	7	37.8	0.19	61.3% (7.4%; 83.8%)	0.024
**Infant mortality** (first 12 months of life)	35	472.0	0.07	23	482.5	0.05	35.2% (−9.6%; 61.7%)	0.102

SP = sulphadoxine-pyrimethamine. ITN = insecticide-treated net. PYAR = person-years at risk.

Table 3 presents the univariate Cox regression analysis of the potential risk factors for neonatal mortality The analysis shows that low birth weight, prematurity, and the presence of parasites in placental tissue (active placental infection) or cord blood, were associated with an increased risk of neonatal death, while IPTp with SP was associated with a reduction of neonatal mortality. The multivariate Cox regression analysis adjusted by treatment group is presented in table 4. In this analysis, the significance of the association of the variables associated with an increased risk is maintained, while the effect of IPTp with SP is not longer statistically significant [Hazard Ratio = 0.56 (95% CI 0.22, 1.43) p value = 0.23]. The increased risk for neonatal mortality of the category of unknowns in some of the variables is likely to reflect the increased risk associated with delivering outside the health facility.

**Table 3:** Univariate and multivariate analysis of the risk factors for neonatal mortality.

N = 970		n	%	HR	(95% CI)	p
**Placental malaria**	**Not infected**	424	43	1.00		0.010
	**Past infection**	321	32	0.82	(0.27–2.52)	
	**Active infection**	124	12	3.92	(1.51–10.15)	
	**Unknown**	128	13	1.25	(0.33–4.71)	
**Cord blood parasitaemia**	**No**	825	83	1.00		0.005
	**Yes**	9	1	10.93	(2.53–47.34)	
	**Unknown**	163	16	1.80	(0.71–4.56)	
**Low birth weight**	**No**	872	87	1.00		<0.001
(<2500g)	**Yes**	116	12	10.16	(4.46–23.18)	
	**Unknown**	9	1	21.76	(4.76–99.35)	
**Pre-term birth**	**No**	907	91	1.00		<0.001
(<37weeks)	**Yes**	40	4	21.13	(7.66–58.29)	
	**Unknown**	50	5	28.09	(10.06–64.67)	
**Gravidity**	**Primigravidae**	256	26	1.00		0.484
	**1 to 3 pregnancies**	395	40	0.57	(0.22–1.49)	
	**4 or >pregnancies**	346	35	0.66	(0.25–1.70)	
**HIV test**	**Negative**	645	65	1.00		0.337
	**Positive**	197	20	1.09	(0.43–2.75)	
	**Unknown**	155	16	0.23	(0.03–1.72)	
**RPR Syphilis test**	**Positive**	116	12	1.00		0.194
	**Negative**	881	88	0.52	(0.20–1.39)	
**Literacy**	**Read and/or write**	410	41	1.00		0.406
	**Not read and write**	584	59	1.82	(0.76–4.35)	
**Newborn gender**	**Male**	513	51	1.00		0.977
	**Female**	480	48	0.99	(0.45–2.17)	
**Foetal anaemia**	**No**	764	77	1.00		0.325
(PCV<37% in cord blood)	**Yes**	69	7	2.07	(0.60–7.09)	
	**Unknown**	164	16	1.76	(0.69–4.49)	
**Malaria episodes during pregnancy**	**No**	908	91	1.00		0.213
	**Yes**	89	9	1.97	(0.68–5.74)	
**Treatment group in the IPTp trial**	**Placebo**	500	50	1.00		0.033
	**SP**	497	50	0.39	(0.16–0.93)	

HR = Hazard Ratio. CI = Confidence Interval. p = p-value Fisher's exact test.

Past infection refers to the presence of malaria pigment but not parasites in the histology.

Active infection refers to acute infection (presence of malaria parasites and minimal pigment) and chronic infection (presence of malaria parasites and pigment) in the histology.

Unknown HIV test = refused voluntary counselling and testing. RPR test = rapid plasma reagin test.

**Table 4:** Multivariate analysis of the risk factors for neonatal mortality.

		Placebo group			SP group					
		(N = 500)				(N = 497)		Hazard		
		n	N	%	n	N	%	Ratio	(95% CI)	p
**Placental malaria**	**Not infected**	5	211	2.4	3	213	1.4	1.00		0.002
	**Past infection**	4	140	2.9	1	181	0.6	0.94	(0.29–3.02)	
	**Active infection**	6	78	7.7	3	46	6.5	5.02	(1.79–14.03)	
	**Unknown**	3	71	4.2	0	57	0.0	0.38	(0.06–2.46)	
**Cord blood parasitaemia**	**No**	13	413	3.1	4	412	1	1.00		0.027
	**Yes**	1	5	20	1	4	25	8.48	(1.72–41.80)	
	**Unknown**	4	82	4.9	2	81	2.5	0.76	(0.19–2.96)	
**Low birth weight**	**No**	6	436	1.4	4	436	0.9	1.00		0.020
(<2500g)	**Yes**	10	59	16.9	3	57	5.3	3.70	(1.37–9.99)	
	**Unknown**	2	5	40	0	4	0.0	4.35	(0.62–30.55)	
**Pre-term birth**	**No**	6	448	1.3	2	459	0.4	1.00		<0.001
(<37weeks)	**Yes**	4	22	18.2	3	18	16.7	6.42	(1.90;21.68)	
	**Unknown**	8	30	26.7	2	20	10	38.85	(11.85;127.34)	

Hazard Ratio of neonatal death adjusted by IPTp intervention group.

## Discussion

Almost all (99%) of the 4 million annual neonatal deaths arise in countries where establishing the causes of death is hampered by limitations in the quality and quantity of the sources of information. Causality of neonatal deaths is thus, mainly based on estimations and most causes of death are unknown. [3] In endemic countries, maternal malaria has been said to contribute to neonatal mortality indirectly through low birth weight and prematurity, though, to our knowledge, no strong evidence exists to support this association. [5] The results of this randomised, double blind, placebo-controlled trial showed that, maternal malaria may have a direct effect on neonatal mortality, and that prevention of malaria during pregnancy can reduce neonatal mortality, on average, by 60%. This information is of public health relevance for malaria endemic countries in Africa, and should serve to stimulate at national and international levels, the implementation of malaria prevention strategies in pregnancy, not only as a measure to reduce low birth weight but also to directly reduce neonatal mortality.

An effect of malaria prevention in pregnancy on perinatal and neonatal survival has been suggested previously. Some trials of malaria preventive strategies in pregnancy (chemoprophylaxis, IPTp or ITNs), showed individually a trend towards a reduction in perinatal death or fetal loss, [11], [12], [23], [24], [25], [26] which became significant when a meta-analysis was carried out. [27], [28] However, a significant reduction on neonatal deaths was not shown in the meta-analysis of the three trials reporting this outcome [overall Risk Ratio 0.68 (95% CI 0.44; 1.05)]. [12], [14], [23] Two of these trials used IPTp with SP but unlike the current study in which ITNs were given as part of the study, ITN coverage was low in the other two studies. [14], [23] As it has been suggested for infants, it could be speculated that the difference in the effect on neonatal mortality between the current study and these previous two reports might be explained by the synergistic effect of ITNs and IPTp. [29]

Not surprisingly, most (80%) of the neonatal deaths occurred within the first week of life. The effect of the intervention on neonatal survival seemed to be concentrated within this short period after birth, where two thirds of the deaths occurred among the babies born to women in the placebo group. The physiopathological mechanisms that may explain the significant protective efficacy of two doses of SP given intermittently in reducing neonatal mortality are not clear. An increase in birth weight or gestational age is unlikely to be the explanation since both outcomes were shown to be similar between the two study groups. [15] On the other hand, the presence of parasites in the placenta or in cord blood may have had a negative effect on neonatal survival. IPTp with SP was significantly associated with a reduced risk of neonatal mortality in the univariate analysis of risk factors but not in the multivariate model, where placental malaria, low birth weight, cord blood parasitaemia and pre-term birth had a stronger association with the risk of death. This indicates that the effect of the intervention is probably due to a reduction on placental and cord blood infection as these variables are in the causal pathway between the effect of the intervention and death. The local inflammatory reactions associated with active placental infection may lead to a reduction in the transfer of nutrients to the foetus, while cord parasitemia may induce fetal anemia and this in turn may lead to hypoxia, all of which may contribute to neonatal death. [30] This argument is supported by the finding of fetal anemia, active placental infection and cord parasitemia more frequently in the placebo than in the SP group. [15] Thus, it could be argued that malaria parasitemia in the last weeks of pregnancy may have a negative direct impact on neonatal survival.

A reduced risk of neonatal infections in the intervention group due to the antibiotic effect of SP can not be completely ruled out. It has been reported that infections are an important cause of neonatal death in developing countries. [31] In this study, although most neonatal deaths occurred at home, nearly all of those occurring at the hospital were due to infectious diseases. However, it seems unlikely that SP was effective against the infections most frequently occurring during the neonatal period in this area, mainly Staphilococcus aureus, group B Streptococcus, and E coli, as it has been shown in this area the low sensitivity of other sulpha drugs. [32]

In this area, there was a 21% level of *in vivo P. falciparum* parasitological resistance to SP in children before this study started, which could have increased during the course of the study, thus reducing the efficacy of the drug as antimalarial. [19] However, the observed efficacy in terms of clinical malaria protection in the pregnant women was over 70% within the month after each SP dose. [15] This high clinical efficacy of the intervention in the pregnant women highlights the difficulties in extrapolating results from drug efficacy studies in children to preventive trials and even more in adults. Nonetheless, parasite resistance to SP has spread across Africa and at some point this will undermine the efficacy of IPTp-SP. Therefore, research into new, long half-life antimalarials for IPTp is urgently needed.

In summary, these results confirm a significant effect of malaria prevention in pregnancy in reducing neonatal mortality. Although they are based on small numbers, and thus the role of chance can not be completely ruled out, it can be said that the observed efficacy of IPTp of over 60% is of relevance for the global initiatives aiming at reducing the high burden of neonatal and perinatal mortality. Moreover, the preliminary economic evaluation of this intervention on neonatal mortality shows a cost effectiveness ratio of just 1.08 USD per DALY averted (Sicuri et al in preparation). Thus, malaria prevention in pregnancy through IPTp with SP, and probably in general, may be one of the most cost-effective public health interventions to reduce neonatal mortality.

## Supporting Information

Figure S1 Trial Profile
(0.09 MB TIF)

Protocol S1 Analytical Plan
(0.18 MB DOC)

Attachment/Main trial publication (0.21 MB PDF)

Attachment/Ethical Approval (0.21 MB PDF)

Checklist S1 (0.19 MB DOC)
