# Supplementary figures and images for: Malaria Prevention with IPTp during Pregnancy Reduces Neonatal Mortality
Source: PLoS One. 2010 Feb 26;5(2):e9438. doi: 10.1371/journal.pone.0009438 (PMC2829080; doi:10.1371/journal.pone.0009438)

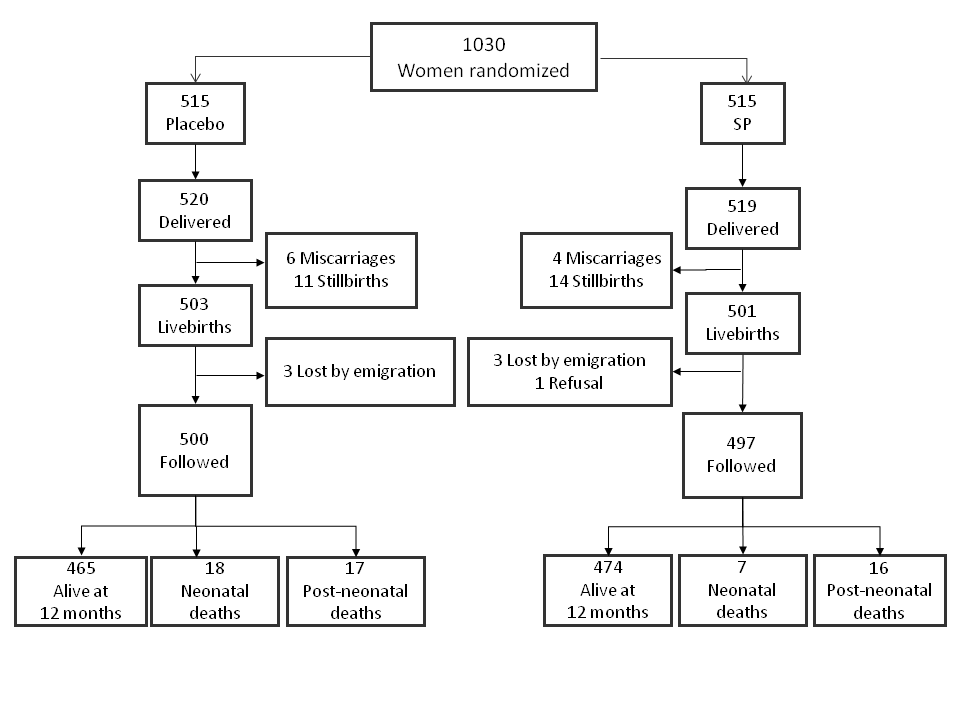

Supplement: Figure S1 — Trial Profile (0.09 MB TIF) [file pone.0009438.s001.tif]
