# Supplementary material for: Malaria Prevention with IPTp during Pregnancy Reduces Neonatal Mortality
Source: PLoS One. 2010 Feb 26;5(2):e9438. doi: 10.1371/journal.pone.0009438 (PMC2829080; doi:10.1371/journal.pone.0009438)
Supplement: Protocol S1 — Analytical Plan (0.18 MB DOC) [file pone.0009438.s002.doc]

# ANALYTICAL PLAN FOR:

# EFFECT OF INTERMITTENT TREATMENT WITH SULFADOXINE-PYRIMETHAMINE PLUS INSECTICIDE TREATED NETS, DELIVERED THROUGH ANTENATAL CLINICS FOR THE PREVENTION OF MALARIA IN MOZAMBICAN PREGNANT WOMEN

TIMNET study

Analytical Plan version 9

Barcelona, 20-01-2005

## CONTENTS

## INTRODUCTION

## 1.Title of the study:

Effect of intermittent treatment with sulfadoxine-pyrimethamine plus insecticide treated nets, delivered through antenatal clinics for the prevention of malaria in Mozambican pregnant women

### 2. Protocol summary:

This document details the methods to be used for the processing and analysis of data collected during the double-blind, placebo-controlled, randomised trial entitled “Effect of intermittent treatment with sulfadoxine-pyrimethamine plus insecticide treated nets, delivered through antenatal clinics for the prevention of malaria in Mozambican pregnant women“. Recruitment to the study started on the 19th of August 2003 in Manhiça, Mozambique, and was completed on the 4th of April 2005. 1030 pregnant women were randomly assigned to receive either two treatment doses of sulfadoxine-pyrimethamine or placebo at the beginning of the second trimester given at least one month apart. All women received a long lasting insecticide treated net (ITN)*. The end of follow-up for each woman is up to two months after delivery. The end of follow up for this analysis is November 15^th^ 2005. Data up this point in time will be included in the analysis.

*** Permanet ® 2.0.** Vestergaard Frandsen

**3. Inclusion criteria**

All women from the study area with:

- less than 29 week of gestational age
- no allergies to sulpha drugs
- informed consent signed

**4. Criteria for administration of the intervention SP/Placebo**

- 1^st^ dose : being at least 12 weeks of gestational age and less than 29 weeks of gestational age at the time of recruitment
- 2^nd^ dose: at least four weeks from the first dose of SP or placebo

**5. Sample size estimation**

Assuming a proportion of low birth weight babies of 20% in the absence of malaria control measures and a 25% reduction in this figure by the use of ITNs to 15%, 411 pregnant women in each intervention group will be requires to show a lack of difference between the two groups with a confidence interval of 7 with at the 5% Level of significance and with a statistical power of 80% , according to he formula for equivalent studies (1) (see references).

n= (2p (100-p)/d^2^) x f (α, β).

where p is the expected proportion; d is the width of the interval; f is a function that depends on the power, thus for α= 0.05 and β= 0.2 is 7.9

PRIMARY ENDPOINT AND SECONDARY ENDPOINTS

## The primary objective of the trial is to evaluate the impact of two doses of intermittent treatment with sulfadoxine-pyrimethamine on low birthweight prevention, in women who had received an ITN through the antenatal clinic.

In addition to this endpoint, different comparisons between the SP and placebo groups will be made. The list is presented according to the group of samples required to measure each outcome.

#### Group 1. Children born alive with birthweight registered

Primary outcome (Prevalence of low birthweight)

1. Birthweight

#### Group 2. Women with peripheral blood samples at delivery

1. Prevalence of maternal peripheral *P falciparum* asexual parasitaemia
2. Density of maternal peripheral *P falciparum* asexual parasitaemia in those parasite positive
3. Prevalence of severe maternal anaemia by PCV
4. Prevalence of overall maternal anaemia by PCV
5. Prevalence of severe maternal anaemia by Hb
6. Prevalence of overall maternal anaemia by Hb
7. PCV
8. Hb

#### Group 3. Cord blood samples

1. Prevalence of foetal anaemia by PCV in cord blood
2. Prevalence of foetal *P falciparum* parasitaemia in cord blood
3. Density of foetal *P falciparum* asexual parasitaemia in cord blood
4. PCV in cord blood

#### Group 4. Placental samples

1. Prevalence of any placental malaria infection (by either histology or impression smear)
2. Proportion of type of placental malaria infection (no infection, acute active infection, chronic active infection and past infection)

#### Group 5. Newborn peripheral blood

1. Prevalence of anaemia by PCV
2. Prevalence of *P falciparum* parasitaemia
3. Density of *P falciparum* asexual parasitaemia
4. PCV

#### Group 6. Newborn with gestational age measured by the Dubowitz method

1. Prevalence of prematurity
2. Mean gestational age

#### Group 7. Women with blood sample at Cross Sectional Visit 1(CSV1)

1. Prevalence of severe maternal anaemia by PCV
2. Prevalence of overall maternal anaemia by PCV
3. PCV
4. Prevalence of maternal peripheral *P falciparum* asexual parasitaemia
5. Density of maternal peripheral *P falciparum* asexual parasitaemia in those parasite positive

#### Group 8. Children with blood sample at CSV1

1. Prevalence of overall anaemia by PCV
2. Prevalence of malaria parasitaemia
3. Density of peripheral *P falciparum* asexual parasitaemia

#### Group 9. Morbidity surveillance during pregnancy

1. Time to first or only episode of clinical malaria
2. Time to first or only episode of severe malaria
3. Time to first or only episode of overall anaemia by PCV
4. Time to first or only episode of severe anaemia by PCV
5. Time to first or only episode of hospital admission
6. Incidence of hospital admissions with anaemia
7. Incidence of all hospital admissions
8. Incidence of outpatients visits

#### Group 10. Morbidity surveillance during post-partum period

1. Time to first or only episode of clinical malaria
2. Time to first or only episode of overall anaemia
3. Time to first or only episode of severe malaria
4. Time to first or only episode of hospital admission during pregnancy and post-partum period
5. Time to first or only episode of hospital admission with malaria
6. Incidence of hospital admissions with anaemia
7. Incidence of hospital admissions with malaria
8. Incidence of all hospital admissions
9. Incidence of outpatients visits

#### Group 11. Morbidity surveillance in children’s OPD

1. Incidence of all cause hospital admissions in study children until the 2nd moth of age
2. Incidence of all cause outpatient visits in study children until the 2nd month of age

#### Group 12. Other endpoints

1. Prevalence of the use of ITN during pregnancy
2. Prevalence of the use of ITN after delivery
3. Prevalence of vomiting after each intermittent treatment
4. Proportion of women with any skin reactions within 27 days from having received the dose
5. Proportion of women with skin reactions by severity within 27 days from having received the dose
6. Proportion of maternal deaths
7. Prevalence of miscarriage
8. Proportion of perinatal deaths (stillbirth and early neonatal death)
9. Proportion of late neonatal deaths
10. Proportion of infant deaths

## ANALYSIS COHORTS

Two analysis cohort are defined:

The Intention to treat (ITT) cohort include all women randomized in the trial and from whom data is available for the analysis

The According to protocol (ATP) cohort include all women who fulfil all the inclusion-exclusion criteria and took the two IPT/Placebo doses and from whom data is available for the analysis

The primary analysis will be done *by intention to treat*,

During the study follow-up two different types of withdrawals will be considered:

A *study withdrawal* will be:

- any pregnant women refusing to continue in the study, as well as their infants
- any woman who migrated out of the study area more than three months,
- women who did not come to the postpartum visit (drop out).

A *treatment withdrawal* will be any pregnant women presenting any adverse effects due to the intervention. In this case the woman will continue taken part of all study procedures (and analysis of the ITT cohort but not of the ATP) and follow up except that she will not receive further interventions

## STATISTICAL METHODS AND MOCK TABLES

### Trial Profile

A trial profile will be done documenting the number of women invited to participate in the study, the number of giving informed consent and therefore randomised, the number of giving an ITN, the numbers randomised to receive either SP or placebo, the number of registered deliveries, the number of registered visits after delivery, and the number completing follow-up until 2 months after delivery. The numbers of withdrawals and deaths up to dose 1of SP/placebo, between doses 1 and 2, and between dose 2 until after delivery will also be included, as well as the number of deliveries by place (See the following mock graph of the trial profile).

XX Study area women

XX randomized

Women not eligibles:

- X gestational age >28 w

- X allergies to sulfonamides

- X refusals

XX received ITN

XX randomized

to Placebo

XX randomized

to SP

XX received 1^st^ dose Placebo

XX received 1st dose

SP

- X migrations

- X withdrawals

- X deaths

XX received 2nd dose Placebo

XX received 2nd dose SP

- X migrations

- X withdrawals

- X deaths

- X migrations

- X withdrawals

- X deaths

XX Deliveries

-BW registered

XX Deliveries

- BW registered

- X live births

- X stillbirth

- X miscarriage

- X twins

- X live births

- X still birth

- X miscarriage

- X twins

- X migrations

- X withdrawals

- X deaths

- X migrations

- X withdrawals

- X deaths

- X migrations

- X withdrawals

- X deaths

Annex 1. Trial Profile

Birthweight included in the analysis are those registered at Manhiça District Hospital with digital weight scale.

Some 1^st^ doses on the second trimester may be not done at the time of receiving the ITN due to malaria concomitant treatment.

### Baseline characteristics

Baseline characteristics (e.g. at randomisation) of SP and placebo recipients will be compared. Variables included in this analysis will be:

- Maternal age

- Gestational Age (mean and by category, at recruitment)

First trimester 0-12 weeks

Second trimester 13-24 weeks

Third trimester > or = 25 weeks

- Parity (mean and by category)

Primigravidae (no previous pregnancy)

1 to 3 previous pregnancies

4 or more previous pregnancies

- Region

- HIV status

- MUAC (Mid-upper arm circumference) index

- Literacy (read and/or write)

- Previous visits to the ANC before the recruitment

- Height (cm)

- RPR results at 1^st^ antenatal clinic

- Hb results at 1^st^ antenatal clinic (g/dl)

In assessing comparability, more emphasis will be given to the size of any differences than to statistical significance, as well as to the relationship with the outcome, since this is what affects the degree of confounding.

### Analysis of prevalence and dichotomic outcomes

A crude 2x2 frequency tables will present the categories and treatment received at dose 1 with the percentage of each category according to the total of subjects in that group

Stratified tables by parity and HIV status at baseline are presented

The effect of the SP will be evaluated using the Protective Effect (PE) defined as 1- RR. The Relative Risk, 95% confidence interval and p-value will be estimated using a modified Poisson regression approach as described by Zou (2). Crude estimates adjusted by maternal age, gestational age, parity, region and HIV status will be presented.

|  | SP | Placebo | RR (95% IC) P-value |
| --- | --- | --- | --- |
|  | n / N (%) | n / N (%) |  |
| Crude |  |  |  |
| By parity |  |  |  |
| In primigravidae |  |  |  |
| In 1-3 prev preg |  |  |  |
| In 4 or + prev preg |  |  |  |
| By HIV |  |  |  |
| In HIV positive |  |  |  |
| In HIV negative |  |  |  |
| In HIV Unknown |  |  |  |

|  | RR | (95% CI) | P-value |
| --- | --- | --- | --- |
| Crude |  |  |  |
| Adjusted by parity |  |  |  |
| Adjusted by HIV status |  |  |  |
| Adjusted by maternal age |  |  |  |
| Adjusted by gestational age |  |  |  |
| Adjusted by region |  |  |  |

### Analysis of non dichotomic proportions

A 2 x n frequency table will be presented. The homogeneity of the distribution of the categories will be compared using the Fisher’s exact test.

### Analysis of the continuous variables

For the density: a table of the geometric mean and IQ range will be presented. For the others: the mean and SD. The effect of the treatment will be evaluated using the Wilcoxon rank-sum test.

### Analysis of the Time to outcomes:

During pregnancy: from 1^st^ dose till till delivery, cross sectional 1,death or migration, or the outcome.

During post-partum period: from delivery till cross sectional 1, death, migration or the outcome. If there is no cross sectional 1 the maximum time will be sixty days.

During pregnancy and post-partum, from those

Failure graphs will be presented using the methodology described by Kaplan-Meier.

A crude table with the number of subjects that have at least one episode and the time at risk by group will be present as well as tables stratified by parity and HIV status.

The effect of the SP will be evaluated using the Protective Effect (PE) estimated as 1-HR. The Hazard Ratio (HR) will be estimated using Cox proportional regression models. Crude and adjusted by parity, adjusted by HIV status, and adjusted by all baseline covariates will be presented.

### Analysis of incidence endpoints

Incidence will be calculated for each group as the number of episodes over the time at risk. A crude table with the number of events and the time at risk as well as tables stratified by parity and HIV status. The effect of the SP will be evaluated using the Protective Effect (PE) estimated as (1-RR) where the Relative Risk (RR) is estimated using Poisson regression models. For endpoints where there could be more than one event per subject, a normal random effect Poisson model will be used to take into account the possible extra-poisson variation due to the non-independency of the events.

DEFINITIONS

1. Severe maternal anaemia by PCV: Haematocrit below 21% or Hb below 7g/dl during pregnancy (3).
2. Overall anaemia by PCV: Haematocrit less than 33% or Hb below 11g/dl, in both mother and infant (4).
3. Severe anaemia in the infant by PCV: Haematocrit less than 25% .
4. Foetal Anaemia by PCV: Haematocrit less than 37% in cord blood and/or Htc<42% in newborn peripheral blood
5. Congenital malaria: the presence of asexual *P falciparum* parasites of any density in a blood smear or detected by PCR in a filter paper sample in cord or newborn peripheral blood until the 7^th^ day of life
6. Malaria infection: the presence of asexual *P falciparum* parasites of any density in a blood smear or detected by PCR in a filter paper sample.
7. Placental infection: According to the histological evaluation, (5)
   1. *acute infection*: parasites present, with absent or minimal pigment deposition within fibrin or cells within fibrin or any amount of fibrin in free macrophages *
   2. *chronic infection*: presence of parasites and a significant amount of pigment deposition in fibrin or cells within fibrin,
   3. *past infection*: presence of pigment with absence of parasites.

All cases with parasites (acute and chronic) were called *active infections*.

1. Low birth weight: less than 2500g (up to and including 2499g) (6).
2. Premature birth: birth before the beginning of the 37th week (7).
3. Fever: an axillary temperature equal or greater than 37.5ºC.
4. Maternal clinical malaria: malaria infection plus any signs and/or symptoms suggestive of malaria disease as referred history of fever in the last 24 hours, axillary fever (Tº>=37.5 ºC), pallor, arthromyalgias, headache and history of convulsions.
5. Maternal severe malaria: positive peripheral parasitaemia and at least one of the following signs or symptoms;
   1. severe anaemia (as defined in definition 1),
   2. cerebral malaria: Glasgow Coma Scale<15, convulsions, and/or coma,
   3. hypoglycaemia: glycaemia < 2.6 mmol/L
   4. respiratory distress: respiratory rate > 40 br/min
   5. jaundice
   6. haemoglobinuria: presence of red blood cells on the urine
   7. vomiting
   8. high parasitaemia will be considered > or = 20.000 parasites/nlitre
6. Maternal non Severe malaria: a malaria infection episode without none of the clinical criteria described in the above definition
7. According to Protocol analysis (ATP): the analysis includes the pregnant women who received an ITN and the two doses of intermittent treatment or placebo within the predefined intervals.
8. Intention to treat analysis (ITT): the analysis includes all recruited women.

16. Not infected placenta: no evidence of parasites or pigment can be identified by histological assessment (5)

17. Cross sectional visit 1 (CSV1): Follow up visit than can be done within sixty days after delivery

18. HIV status: Human immunodeficiency virus status test is done following the National Programme for HIV/AIDS Control guidelines; Voluntary Counseling and Testing test with Determine® reagents as first choice and an Unigold® reagents in case of indetermined result.

19. MUAC: Mid-upper arm circunference: Normal range: plus than 22 cm. Indirect index of malnutrition in adults.

20. RPR: Rapid Plasma Reagin test; non treponemal test.

* Any amount of pigment on free macrophages is not included in reference 5.

## DATA MANAGEMENT AND BREAKING THE RANDOMISATION CODE

Data has been double-entered by two different data entry clerks using a menu driven system gratin in Visual FoxPro 5 run over a Windows NT network (Data management for Field Trials v2). All data was stored and processed in a dedicated secure directory on a central server. A data manager performed daily cross-checking routines to detect and correct any discrepancies between the two entries. Discordances detected at this point were recorded in a log file permitting quality control of the data checking process. Weekly checks for duplicate records, completeness of the databases, range, consistency and referential integrity were also performed.

The cleaned and locked database files will be handed to the trial PI and co-Pis in exchange for the randomisation code. As extended follow-up until 12 months will continue after the first analysis, the field team will remain blind to avoid compromising the analysis till then. The first analysis will proceed as follows.

## KEY DATES FOR DATA ANALYSIS

Last delivery: 7 October 2005

Last postpartum visit: 7 November 2005

Lock of databases: 7 December 2005

**Data bases**

| TNRECRU | Reclutamento mulheres |
| --- | --- |
| **TN_NET** | Entrega da rede |
| TNMUAC | Avaliação estado nutricional |
| **TNFIELD** | Visita ao campo |
| **TN_DOSE1** | Administração 1ª dose |
| **TNDOSE2** | Administração 2ª dose |
| **TNDAE** | Efeitos adversos dermatológicos |
| **TNECO** | Estudo socioeconómico |
| **TN_OPD** | Triagem de gravidas/puérperas |
| **TN_INPD** | Internamento de maternidade |
| **TNCHIDTH** | Morte de crianças do estudo |
| **TNMOTDTH** | Morte de mulheres do estudo |
| **TNX1CHI** | Visita 1 mes crianças |
| **TNX1MOT** | Visitas 1 mes maes |
| **TN_PHOTO** | Inquerito foto cartões |
| **TNOUTCOM** | Parto |
| **TNPOSPAR** | Entrevista post-parto |
| **TNIMP** | Impronta placenta |
| **TNHIST** | Histología placentas |
| **TNNEWBOR** | Recem nascidos |
| **TNX12MON** | Visita 12 meses |
| **TNWITHD** | Retiradas do estudo |
| **TIM_HEMA** | Hemogramas |
| **READINGS** | Leituras definitivas láminas |
| **HTOLONG** | Resultados de hematocrito |
| **SAMPLES** |  |
| **SAMPLES2** | Posicionamento |
| **POSPFILT** | Posicionamento papel de filtro |
| **RAPFILT** | 1,2,3 Posicionamento papel de filtro del parto |
| **RESHAPE_HTO** |  |
| **RESHAPE_SLIDEPOS** |  |

**References**

1. Pocock SJ 1993. Clinical trials: A practical approach
2. Zou et al, American Journal of Epidemiology, 2004; 159:702-706
3. WHO. 1992. The prevalence of anaemia in women. WHO/MCH/MSM.92.2 Geneva
4. Iron deficiency in the Tropics. Fleming. Clinics in Haematology.1982;11,2
5. Mamudo R. et al, Human Pathology, 2000; 31:85-93
6. Low birthweight. Country, regional and global estimates. WHO. Unicef. 2004
7. Berkowitz GS, Epidemiologic Reviews. 1993; 15: 414-43
