# Supplementary material for: Malaria Prevention with IPTp during Pregnancy Reduces Neonatal Mortality
Source: PLoS One. 2010 Feb 26;5(2):e9438. doi: 10.1371/journal.pone.0009438 (PMC2829080; doi:10.1371/journal.pone.0009438)
Supplement: Attachment/Ethical Approval — (0.21 MB PDF) [file pone.0009438.s004.pdf]

REPÚBLICA DE MOÇAMBIQUE
MINISTÉRIO DA SAÚDE

COMITÉ NACIONAL DE BIOÉTICA PARA A SAÚDE

Ex^{mos} Senhores
Centro de Investigação
em Saúde da Manhiça

Ref: 017/CNBS/03 **Data:** 14 de Maio de 2002

Assunto: Aprovação do Comité Nacional de Bioética para a Saúde (CNBS) sobre o protocolo referente ao tratamento intermitente com SP e redes mosquiteiras.

Reunido no dia 26 de Abril de 2003 Comité Nacional de Bioética para a Saúde (CNBS) analisou o protocolo com o seguinte título **"Efeito do tratamento intermitente com sulfadoxina-pirimetamina com redes mosquiteiras impregnadas, realizado através das consultas pré-natais na prevenção da malária em mulheres grávidas de Moçambique"** e, sobre o mesmo, somos a informar que não existe nenhum inconveniente de ordem ética que impeça a realização do estudo. Assim, o CNBS dá a devida autorização para a sua realização.

Recomendamos que os investigadores mantenham o CNBS informado do decurso do estudo.

Sem mais assunto, os nossos cumprimentos.

Presidente

REPÚBLICA DE MOÇAMBIQUE MINISTÉRIO DA SAÚDE COMITÉ NACIONAL DE BIOÉTICA PARA A SAÚDE

Dr. João Manuel de Carvalho Fumane

ENDEREÇO:
MINISTÉRIO DA SAÚDE
C. POSTAL 264
Av. Eduardo Mondlane/Salvador Allende
MAPUTO – MOÇAMBIQUE

Telefone/Fax: 2581 43 11 03
Telex: 6-239 MISAU MO
